# Attenuation and recovery of an avoidance response to a chemical antipredator cue in an invasive fish: implications for use as a repellent in conservation

**DOI:** 10.1093/conphys/coac019

**Published:** 2022-04-07

**Authors:** C Michael Wagner, Jason D Bals, Mikaela E Hanson, Anne M Scott

**Affiliations:** Department of Fisheries and Wildlife, Michigan State University, East Lansing, MI, 48824, USA; Department of Fisheries and Wildlife, Michigan State University, East Lansing, MI, 48824, USA; Department of Fisheries and Wildlife, Michigan State University, East Lansing, MI, 48824, USA; Department of Fisheries and Wildlife, Michigan State University, East Lansing, MI, 48824, USA

**Keywords:** Habituation, Alarm Cue, Repellent, Lamprey, Invasive Species

## Abstract

The detection of predation risk without direct engagement with a predator is an important driver of prey movement strategies. Consequently, the application of alarm cues may prove an effective tool in guiding the movements of fishes targeted for control or conservation. However, failure to contemplate the sensory, physiological and cognitive outcomes of repeated or persistent exposure to the cue will likely lead to poor performance of management practices. Using a series of behavioural tests and physiological recordings from the olfactory organ, we examined the timing of onset and recovery of the alarm response in sea lamprey (*Petromyzon marinus* L.) when exposed continuously or sporadically to its alarm cue. In the laboratory, sea lamprey exhibited short-term, reversible attenuation of the alarm response over 2–4 h with continuous exposure. The alarm response spontaneously recovered after 30–60 min of removal from the cue. In long-duration free-swimming tests, where the animals were allowed to move into and out of the odour plume volitionally, repeated but sporadic encounter with the alarm cue over 5 h did not alter the alarm response. Electro-olfactogram recordings from the main olfactory epithelium indicated that olfactory sensory neurons quickly adapt to alarm cue and recovered within 15 min. Our findings strongly implicate habituation as the mechanism that induces reduction in the alarm response and provide insight into the design of effective management practices that seek to use fish alarm cues as repellents.

## Teaser

Fear is a powerful motivator of animal movements and can be used as a repellent in conservation activities. Invasive sea lamprey exhibit both sensory adaptation and short-term habituation to its alarm cue when exposed continuously, but not sporadically, suggesting a route towards effective management applications that defeat habituation.

## Introduction

The ability to influence the movement decisions of targeted fishes would improve a number of pervasive conservation problems in aquatic ecosystems. These include increasing passage through barriers to migration (Castro-Santos and Haro, 2010; Moser *et al.*, 2019), preventing entrainment and impingement at water intake structures (Noatch and Suski, 2012), the capture and removal of nuisance species (Sorensen, 2015; Butler *et al.*, 2019) and the protection of endangered species (Farnsley *et al.*, 2018). Individual movement decisions are informed by sensory systems that have evolved to detect cues explicitly associated with fitness-impacting circumstances (i.e. the detection of food, predators and mates). In aquatic ecosystems, this information often takes the form of semiochemicals: substances that either directly communicate (signals) or inadvertently advertise (cues) the immediacy of opportunities or risks. These substances function as potent attractants or repellents and have great appeal for use in conservation, as they typically are taxon specific, and have few unintended negative consequences (Sorensen and Johnson, 2016).

The use of aversive odours associated with the detection and management of predation risk may prove particularly useful in guiding the movements of animals. The risk of predation is rarely absent, yet varies considerably over space and time (Sih *et al.*, 2000). Consequently, the perception of predation risk broadly influences movement decisions, and prey continuously gather sensory information to estimate the immediacy of predator threat (Lima and Dill, 1990; Preisser and Bolnick, 2008; Gaynor *et al.*, 2019; Sabal *et al.*, 2021). Fish alarm cues, substances released from damaged tissue that elicit antipredator behaviour in conspecifics (= *schreckstoff* per von Frisch, 1938, 1941), have been extensively studied (for recent reviews, see Ferrari *et al.*, 2010; Wisenden, 2015). Alarm cues are suspected to be mixtures of compounds, as cross-reactivity is limited to confamilial species, and behavioural responses tend to diminish with increasing phylogenetic distance between the emitter and receiver species (Hazlett and McLay, 2005; Dalesman *et al.*, 2007; Schoeppner and Relyea, 2009; Mitchell *et al.*, 2012; Hume and Wagner, 2018). The compounds that compose the mixture are principally detected by ciliated olfactory sensory neurons (OSNs) that stimulate the medial olfactory tract, inducing avoidance or other antipredator responses (Hamdani and Døving, 2002, 2007; Døving and Lastein, 2009). Perception of the cues can result in persistent avoidance of areas activated by the odour (Wisenden *et al.*, 2009; Chivers *et al.*, 2013).

Introducing an alarm cue to ensure avoidance of an area, or to ‘push’ an animal towards a target, may require sustained application (hours, days or months). For example, guiding migrating fish towards the entrance of a fish passage device may entail temporary exposure of a few hours for each individual, whereas the need to block resident fishes from a water intake would be continuous. However, continuous or frequent exposure to an odour often results in reduced reaction to its presence. Reduction or loss of the avoidance response to alarm cue (hereafter referred to as response attenuation) has been noted in cane toad (*Bufo marinus*; Hagman and Shine, 2009), chub (*Leuciscus cephalus*; Krause, 1993a), dace (*Leuciscus lueciscus*; Krause, 1993b) and sea lamprey (*Petromyzon marinus*; Imre *et al.*, 2017). Diagnosing the sensory and/or cognitive mechanisms underlying the attenuation of the alarm response in a species targeted for manipulation is a necessary step in designing an appropriate application of the cue for conservation activities (Greggor *et al.*, 2014; Blumstein, 2016).

Physiologically, response attenuation may occur in the peripheral sensory system, and/or in any neuron along the sensory pathway, via adaptation (Kurahashi and Menini, 1997; Zufall and Leinders-Zufall, 2000). Olfactory adaptation operates at the receptor, resulting in a loss of signal transduction. Alternatively, or in concert with adaptation, habituation may occur, a process occurring in the brain that is a form of non-associative learning (Wilson and Linster, 2008; Rankin *et al.*, 2009). Habituation typically results in the animal adjusting its response threshold to attenuate the behavioural response to a continuous ‘background’ signal from the sensory organ that is uninformative. If either adaptation or habituation, or both, are operating, the continuous application of an odour to manipulate animal movement would rapidly become ineffective. Evaluating the timing of the onset and recovery of response attenuation should prove instructive in resolving the mechanisms in play. Adaptation of vertebrate OSNs may be short (onset within <5 s exposure, recovery in 25 s) or long (onset after 25 s of exposure, recovery in a few minutes to an hour) (Dalton, 2000; Zufall and Leinders-Zufall, 2000). Onset and recovery of longer duration implicates habituation (onset in minutes to hours, recovery in several minutes to >1 h, or a much as a few weeks; Wilson and Linster, 2008).

In the current study, we examine the onset and recovery of response attenuation to an alarm cue in sea lamprey. The invasive population of sea lamprey in the Laurentian Great Lakes continues to be a significant impediment to restoration of the largest freshwater ecosystem in the world (Brant, 2019). A great deal of research has been directed towards understanding the annual reproductive migration into rivers, as these habitat selection decisions regulate the distribution of larvae throughout the basin, and thereby the cost and extent of the pesticide applications directed at reducing larval populations (Marsden and Siefkes, 2019). During the migration, sea lamprey respond strongly to an alarm cue (Wagner *et al.*, 2011; Bals and Wagner, 2012; Di Rocco *et al.*, 2016; Luhring *et al.*, 2016), that may be used to guide the movements of migrants in rivers towards control devices (e.g. traps; Hume *et al.*, 2015; Hume *et al.*, 2020) or tributaries targeted for pesticide application (Imre *et al.*, 2010; Wagner *et al.*, 2011). As the animal’s migratory movements are nocturnal, the ability to sustain such manipulations throughout the nighttime (8–10 h) is necessary for achieving conservation goals. Further, the partial conservation of alarm cue in lampreys (Hume and Wagner, 2018) suggests similar movement manipulations would be useful for guiding migrating lampreys of restoration concern towards fish passage devices that provide entry into spawning habitats (Moser *et al.*, 2002; Moser *et al.*, 2011; Maitland *et al.*, 2015; Byford *et al.*, 2016; Porter *et al.*, 2017; Moser *et al.*, 2019).

The first objective of our study was to characterize the timing of attenuation and spontaneous recovery of the alarm response in sea lamprey during continuous exposure to the alarm cue. Second, as animals may also habituate to repeated exposures of olfactory cues, we examined the behavioural responses to alarm cue when the animal was allowed to move freely into and out of the odour plume in a laboratory raceway for evidence of habituation (reduced avoidance) or sensitization (increased avoidance). Finally, we examined the olfactory sensitivity to the alarm cue via electro-olfactogram (EOG) recording to gain insight into the magnitude of sensory adaptation in sea lamprey. We discuss the implications of the findings for use of the alarm cues in behavioural manipulations designed to guide the movements of fishes prioritized for conservation or control.

## Materials and methods

### Test subjects

For these experiments, we used 1106 adult migratory (immature) sea lamprey captured in traps from four tributaries that discharge into Lake Huron or Lake Michigan: the Cheboygan, Ocqueoc, Manistee and St. Mary’s Rivers. Experiments 1, 2 and 3 used immature male sea lamprey. Experiment 4 used immature male and female sea lamprey. Immature male and female sea lamprey exhibit a similar avoidance response to alarm cue (Bals and Wagner, 2012). After capture, the animals were transported to the U.S. Geological Survey’s Hammond Bay Biological Station near Millersburg, MI, USA, in aerated tanks. Lampreys were held in 1000-l tanks (250 animals per tank) with continuous water flow from Lake Huron at ambient water temperature (range, 13–17°C) and the natural light schedule. As migrating sea lamprey cease feeding prior to entering rivers, the lampreys did not receive food. Each animal was held for a minimum of 48 h prior to use in experiments and was used in a single trial. Lamprey used for EOG recordings were transported to the University Research Containment Facility at Michigan State University, East Lansing, MI, USA, and held in continuous flow-through tanks supplied with aerated, chilled well water (range, 7–9°C). All experimental procedures were approved by the Michigan State University Institutional Animal Care and Use Committee (AUF nos. 02/11-027-00 and 02/18-025-00).

### Test odours

We used two test odours: a filtered Soxhlet extract from the whole body that contains a damage-released alarm cue that invokes a strong avoidance response in sea lamprey in laboratory and field environments (Wagner *et al.*, 2011; Bals and Wagner, 2012; Hume *et al.*, 2015) and the Soxhlet extraction solvent (50:50, 200 proof ethanol and deionized water, by volume) as a negative control. We obtained the Soxhlet extract from one male (255 g) and one female (192 g) adult migratory sea lamprey, each extracted separately with a 71/60 Soxhlet body connected to a 3-bulb Allihn condenser and a round bottom flask that contained 1 l of extraction solvent. Each apparatus was heated to 75–80°C using a hemispherical mantle and cycled six times (~6 h). After extraction, the material retained in the flask was allowed to cool to room temperature, combined, filtered under light vacuum (GE Healthcare Whatman™ Qualitative filter paper, Grade 4) and stored at −20°C until use.

### Experiment 1. Attenuation of the avoidance response

To assess the behavioural reaction to the alarm cue after varying periods of continuous pre-exposure, we examined space use in a laboratory two-choice test. Subjects were randomly assigned to one of 60 groups of 10 individuals each. Each of 50 groups was pre-exposed to the alarm cue via continuous immersion in a dilute solution of the Soxhlet extract (1 μl l^−1^ in Lake Huron water) for one of five time periods: 0, 60, 120, 240 or 480 min (0 pre-exposure equates to no prior experience with the extraction solvent or the alarm cue extract; *N* = 10 for each time period). Pre-exposure involved continuously pumping the odour into the flow-through holding water to assure the cue did not breakdown during this stage of the experiment. Ten additional groups were tested for response to the extraction solvent without prior exposure (negative control). The behavioural test was performed in two laboratory raceways, each equipped with a collimator to smooth the flow (stacked PVC pipe, 60 cm l, 2.54 cm inner diameter), and block nets to retain the animals in a 5.0 × 1.84 m experimental reach. Each raceway received a continuous flow of fresh Lake Huron water (13–17°C); water depth was maintained at 30 cm with a mean velocity of 1.8 cm sec^−1^. The source water into the raceway is the same as experienced during holding and pre-exposure, ensuring the animals did not experience any sudden change in water condition at any point in the experiment.

All trials were conducted after 22:00 h, as sea lamprey are nocturnal. At the start of a trial, a group of ten lampreys was released into the experimental reach and allowed to acclimate to the raceway conditions for 10 min. After the acclimation period, a stimulus odour was pumped into one-half of the channel width via peristaltic pump (MasterFlex model 7533-20) for 20 min. The stimulus was formulated by mixing 4 ml of a test odour (alarm cue extract or extraction solvent) into 400 ml of Lake Huron water collected from the raceway in a 500 ml Erlenmeyer flask that was continuously stirred with a 2-cm magnetic stir bar. This mixture was pumped into the raceway at a rate of 15 ml min^−1^ to create a 1 μl l^−1^ dilution in the target half of the channel (odour distribution confirmed via dye tests prior to the start of the experiment). The side of the channel receiving the stimulus odour was alternated between replicates for each pre-treatment time (left or right). The raceways were illuminated with infrared light, and separate infrared-sensitive video cameras recorded the position and movements of each animal during the 30 min trial against two lines painted onto the raceway floors that separated each arena into four equal-sized rectangles. At the conclusion of a trial, the subjects were removed from the raceway and returned to a separate holding tank.

### Experiment 2. Spontaneous recovery of the avoidance response

Results from Experiment 1 suggested 240 min of pre-exposure to the alarm cue fully attenuated the response. To establish the spontaneous recovery threshold (timing of reemergence of the avoidance response), we submitted an additional 400 lamprey (40 groups of 10 individuals each) to an attenuation-recovery experiment. Each group was pre-exposed to the dilute alarm cue extract (1 μl l^−1^) for 240 min. After pre-exposure, groups were either moved immediately into the raceway (recovery time = 0 min), or into recovery tanks receiving continuous ‘clean’ Lake Huron water for 30, 60 or 120 min (*N* = 10 for each time period). After the recovery period, each group was subjected to the two-choice test procedure described in Experiment 1 to ascertain whether and when the animals would again avoid the alarm cue. We expected a recovery time of 0 min would result in no avoidance of the alarm cue during the two-choice test.

### Experiment 3. Behavioural effects of protracted activation of an area with the alarm cue

To test whether frequent, sporadic exposure to the alarm cue in the same location would alter the use of space via learning (predict greater avoidance) or response attenuation (predict lesser avoidance), we conducted a protracted (300 min) two-choice test with lampreys that were not pre-exposed to the alarm cue. Lampreys were randomly assigned into 17 groups of 10 individuals each, and subjected to the two-choice test described in Experiment 1 (sans the pre-exposure protocol). During the 5-h trials, either the alarm cue extract (*N* = 7) or the extraction solvent (*N* = 10) was continuously pumped into one side of the raceway and the animal’s movements were recorded onto digital video. At the conclusion of the experiment, we examined the use of space to ascertain whether the tendency of lamprey to occupy the side of the raceway containing the stimulus changed over time and/or as a function of the odour (solvent control vs. alarm cue).

### Experiment 4. EOG recordings

To determine if continuous exposure to dilute alarm cue resulted in olfactory adaption, we followed previously established EOG recording procedures (Buchinger *et al.*, 2020; Siefkes and Li, 2004) (*N* = 6). Briefly, sea lampreys were anaesthetized with 3-aminobenzoic acid ethyl ester (100 mg l^−1^, MS222, Sigma-Aldrich) and immobilized with an injection of gallamine triethiodide (30 mg kg^−1^ of body weight, Sigma-Aldrich). Gills were continuously irrigated with aerated water containing 50 mg l^−1^ MS222 throughout the experiment. The olfactory lamellae were surgically exposed, and the differential EOG response magnitude was recorded using glass capillary borosilicate electrodes filled with 0.4% agar in 0.9% saline and connected to solid state electrodes with Ag/AgCl pellets (Warner Instruments LLC, model ESP-M15N) in 3 M KCl. EOG signals were amplified (NeuroLog system, Digitimer Ltd, model NL102), filtered (low-pass 60 Hz, Digitimer Ltd, model NL125), digitized (Molecular Devices LLC, Digidata 1440A) and recorded on a computer running AxoScope 10.4 software (Molecular Devices LLC).

Stock solutions of _L_-arginine (10^−3^ M) in deionized water, a sea lamprey sex pheromone 3-keto petromyzonol sulphate (3kPZS; 10^−3^ M; Bridge Organics Co.) (Li *et al.*, 2002) in deionized water/methanol (1:1, v:v), and filtered whole body alarm cue extract (Whatman, Puradisc syringe filter 2.0 μm) were prepared, stored at −20°C and then diluted with filtered water to yield working solutions. A baseline EOG response to the blank control, 10^−5^ M _L_-arginine standard, and test stimuli (1 μl l^−1^ alarm cue, 10^−7^ M _L_-arginine, 10^−9^ M 3kPZS; before unadapted responses) were recorded. The test stimuli were used at concentrations that elicited approximately equal magnitude EOG responses. Then, the olfactory epithelium was continuously exposed to 1 μl l^−1^ alarm cue for 15 min. Next, the responses to mixtures of 1 μl l^−1^ alarm cue and 1 μl l^−1^ alarm cue, 1 μl l^−1^ alarm cue and 10^−7^ M _L_-arginine, and 1 μl l^−1^ alarm cue and 10^−9^ M 3kPZS were recorded (during adapted responses). The olfactory epithelium was flushed with charcoal filtered water for 15 min, and responses to the blank control, 10^−5^ M _L_-arginine standard and test stimuli (1 μl l^−1^ alarm cue, 10^−7^ M _L_-arginine, 10^−9^ M 3kPZS; after unadapted responses) were recorded to determine if the olfactory responses recovered after exposure to alarm cue.

### Statistical analyses

All trials in Experiments 1–3 were video-recorded and the videos were analysed during replay. For Experiments 1 and 2, we assigned the position of each animal to the stimulus or non-stimulus side of the raceway every 30 s after the onset of odour introduction for the full 20-min period. To generate the measurement of the proportion of animals on the stimulus side, we summed the counts from each measurement taken during the final 10 min of the trial (20 measures) and calculated the proportion on the stimulus side. For Experiment 3, we analysed every other 10-min period of video beginning 20 min after the onset of odour introduction, using 30-s intervals as previous, similarly generating the proportion on the stimulus side (observation times: 30, 50, 70, 90, 110, 130, 150, 170, 190, 210, 230, 250, 270 and 290 min after the onset of odour introduction). The 10-min intervals between measurement windows were meant to decrease the likelihood of spatial autocorrelation in the measurements during the protracted trials. The data from each experiment were analysed as the proportion of fish on the stimulus side of the raceway, comparing mean proportions across the treatments. Untransformed proportions were examined as the data met the assumption of normality per Shapiro Wilk’s Test (Experiment 1, W = 0.98, *P* = 0.56; Experiment 2, W = 0.98, *P* = 0.54; Experiment 3, W = 0.99, *P* = 0.56), and the assumption of equal variances per Bartlett’s Test (Experiment 1, χ2 = 2.63, *P* = 0.76; Experiment 2, χ2 = 3.62, *P* = 0.31; Experiment 3, χ2 = 1.55, *P* = 0.21).

Data for Experiment 1 were analysed via one-way ANOVA (α =0.05) with pre-exposure time as the main effect (factor, six levels: solvent control and 0, 60, 120 240, 480-min pre-exposure times). A post-hoc Tukey’s HSD test was used to make pairwise comparisons across treatments. In addition, we compared the solvent control result to a nominal 50:50 distribution via one-way *t*-test (α =0.05). Similarly, data from Experiment 2 were examined with a one-way ANOVA (α =0.05) with recovery time as the main effect (factor, four levels: 0, 30, 60, 120-min recovery times). A post-hoc Tukey’s HSD test was again used to make pairwise comparisons across recovery times. Due to limited animals, we did not repeat the extraction solvent control during this experiment. Rather, the data for each recovery time was also subjected to a one-way t-test to compare the mean observed proportion vs. a nominal 50:50 distribution. All analyses for Experiments 1 and 2 were conducted in STATA ver. 14.2 (StataCorp LLC). To examine the data from Experiment 3, we built a mixed effects general linear model with the proportion of animals on the stimulus side as the response, and observation time (factor, 14 levels corresponding to the observation times), treatment (factor, two levels: control or alarm cue), a time-by-treatment interaction and a random effect of the trial (group). The model was then subjected to a Type II ANOVA to test the effects of observation time and treatment on the distribution of animals in the raceway. The model was built and evaluated in R, version 3.5.1, with the nlme package (Pinheiro *et al.*, 2015).

For Experiment 4, the normalized EOG response was calculated for each stimulus before, during, and after alarm cue exposure as Normalized EOG Amplitude = (Rt – Rb)/(Ra – Rb), where Rt is the response magnitude to the test stimulus, Rb is the response magnitude to the blank and Ra is the response magnitude to 10^−5^ M _L_-arginine. The change in the olfactory response to alarm cue, _L_-arginine and 3kPZS during exposure to alarm cue was analysed via a one-way repeated measures ANOVA (α =0.05) with time as the main effect (factor, three levels: before, during and after exposure) in R (version 4.1.0) with the rstatix package. Post-hoc paired *t*-test comparisons with Bonferroni adjustments were used to make pairwise comparisons across time points within a stimulus. The data met the assumptions of normality distributed for each stimulus at each time point as assessed by Shapiro–Wilk’s test (*P* > 0.05) and sphericity as assessed by Mauchly’s test (Alarm cue, W = 0.678, *P* = 0.459; _L_-arginine, W = 0.310, *P* = 0.096; 3kPZS, W = 0.872, *P* = 0.761).

To determine if continuously exposing the olfactory epithelium to alarm cue reduced the response to the three test stimuli to the same extent, we first calculated the percent of the average normalized, blank corrected unadapted responses, defined as the Percent of Unadpated Response = [(R_adapt_ –Rb)/(Ra – Rb)]/[(R_unadapt_ – Rb)/(Ra – Rb)] * 100. Rb is the response magnitude to the blank, and Ra is the response magnitude to 10^−5^ M _L_-arginine. For example, to calculate the change in olfactory response of 3kPZS, R_adapt_ was the response to 10^−9^ M 3kPZS during alarm cue exposure relative to the average of the response to 10^−9^ M 3kPZS recorded before and after exposure (R_unadapt_). Then, the percentage of unadapted response data were analysed via one-way ANOVA (α =0.05) with test stimuli as the main effect (factor, three levels: alarm cue, _L_-arginine, and 3kPZS). A post-hoc Tukey’s HSD test was conducted to make pairwise comparisons across treatments. The percentage of unadapted response data met the assumption of normality per Shapiro Wilk’s Test (Alarm cue, W = 0.80, *P* = 0.06; _L_-arginine, W = 0.89, *P* = 0.33; 3kPZS, W = 0.94, *P* = 0.68) and the assumption of equal variances per Bartlett’s Test (χ^2^_(2)_ = 3.25, *P* = 0.20).

## Results

### Experiment 1. Attenuation of the avoidance response

**Figure 1:**
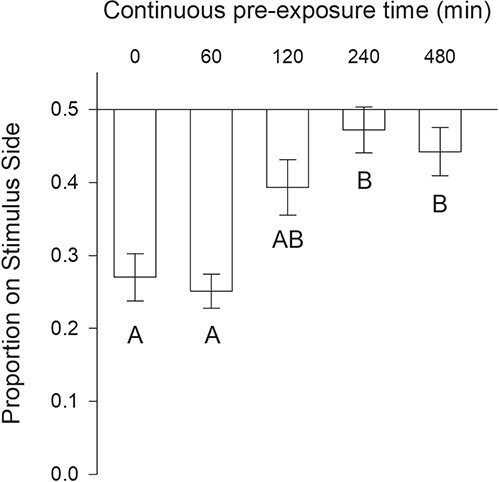
Mean ± SE proportion of lamprey on the stimulus side after continuous pre-exposure to alarm cue during Experiment #1. Time treatments with the same letter are not significantly different from each other 671 (ANOVA with a Tukey Test, α = 0.05). Both the 240- and 480-minute treatments were not significantly 672 different from an equal proportion (*P* > 0.05).

The aim of the first experiment was to determine the timing of the onset of response attenuation during continuous exposure to a fixed dilution of the alarm cue. ANOVA revealed a significant effect of pre-exposure time on the avoidance response to the alarm cue (F_(5,59)_ = 11.12, *P* < 0.001; Fig. 1). Avoidance of the alarm cue began to attenuate after 120 min of pre-exposure, and was fully attenuated after 240 min of pre-exposure. Per Tukey’s HSD pairwise comparisons, the avoidance of the alarm cue was significantly greater than the control only for the 0 min (*t* = 5.03, *P* < 0.001) and 60 min (*t* = 5.47, *P* < 0.001) pre-exposure times. At 240 and 480 min the response did not differ from control (240 min, *t* = 2.22, *P* = 0.25; 480 min, *t* = 1.09, *P* = 0.88) and was significantly less than the avoidance observed after 0 min (240 min, *t* = 4.62, *P* < 0.001; 480 min, *t* = 3.94, *P* = 0.003) and 60 min (240 min, *t* = 5.06, *P* < 0.001; 480 min, *t* = 4.38, *P* = 0.001) of pre-exposure. Avoidance after 120 min, the pre-exposure time intermediate to the fully responding (0 and 60 min) and fully attenuated (240 and 480 min) periods, was significantly less than the 60-min treatment (*t* = 3.26, *P* = 0.023), but differed from no other treatment or the control (Con, *t* = 2.22, *P* = 0.25; 0 min, *t* = 2.82, *P* = 0.07; 240 min, *t* = 1.80, *P* = 0.047; 480 min, *t* = 1.12, *P* = 0.87).

### Experiment 2. Spontaneous recovery of the avoidance response

**Figure 2:**
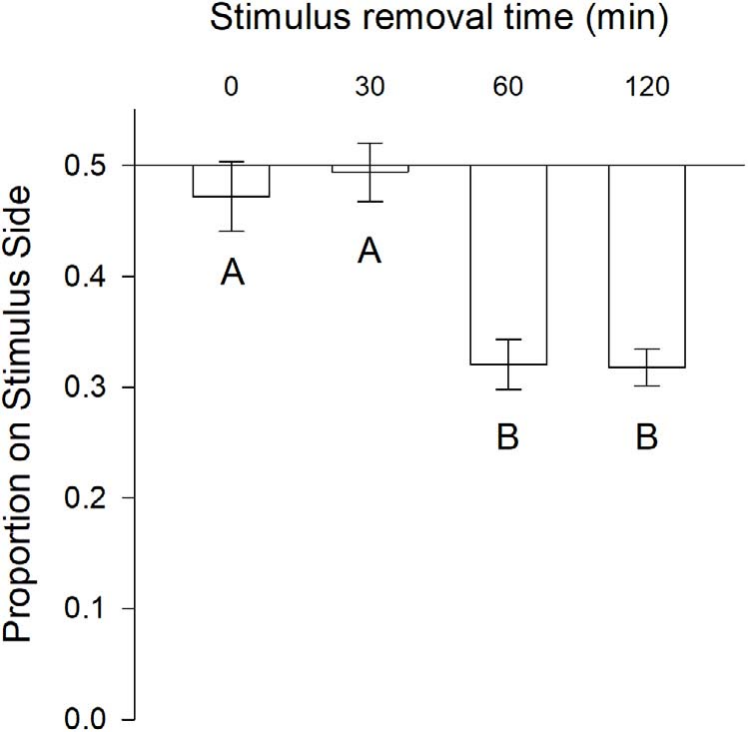
Mean ± SE proportion of lamprey on the stimulus side (alarm cue) after stimulus was removed during Experiment #2. Prior to removing the stimulus, lampreys were exposed to alarm cue for 240 minutes. Treatments with the same letter are not significantly different from each other. (ANOVA with Tukey Test α = 0.05). Both the 60- and 120-minute stimulus removal treatments were significantly different from an equal distribution (*P* < 0.001).

The aim of the second experiment was to determine the timing of spontaneous recovery of the avoidance response to alarm cue. ANOVA revealed a significant effect of recovery time on avoidance of the alarm cue (F_(3,39)_ = 14.55, *P* < 0.001; Fig. 2). Post-hoc comparisons (Tukey’s HSD) revealed significant differences in avoidance behaviour after 60 min (0 vs. 30 min, *t* = 0.62, *P* = 0.93; 0 vs. 60 min, *t* = −4.30, *P* = 0.001; 0 vs. 120 min, *t* = −4.38, *P* = 0.001; 30 vs. 60 min, *t* = −4.92, *P* < 0.001; 30 vs. 120 min, *t* = −5.00, *P* < 0.001; 60 vs. 120 min, *t* = −0.07, *P* = 1.00). Subsequent *t*-tests confirmed no difference from a nominal 1:1 distribution in the raceway after 0 and 60 min of recovery time (*t*-test vs. 1:1 distribution: 0 min, *t*_(9)_ = −0.89, *P* = 0.20; 60 min, *t*_(9)_ = −0.23, *P* = 0.41). However, at 60 min (*t*_(9)_ = −7.93, *P* < 0.001) and 120 min (*t*_(9)_ = −11.07, *P* < 0.001), the lampreys significantly avoided the alarm cue.

### Experiment 3. Behavioural effects of protracted activation of an area with the alarm cue

**Figure 3:**
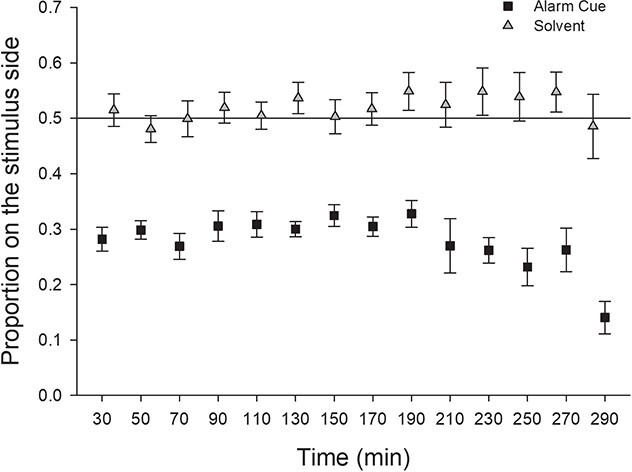
Mean ± SE proportion of lamprey on the stimulus side (alarm cue or solvent) when one-half of the raceway was activated with the stimulus during Experiment #3. Treatment had a significant effect in the model (*P* < 0.001).

The aim of the third experiment was to ascertain whether the avoidance of an area activated with the alarm cue would change over a 5-h time period (Fig. 3). Type II ANOVA on the mixed-effects GLM coefficients revealed a persistent avoidance of the alarm cue vs. ethanol control: (χ^2^ = 31.82, *P* < 0.001), with no effect of observation time (χ^2^ = 1.52, *P* = 0.22), nor evidence of a time-by-odour interaction (χ^2^ = 1.42, *P* = 0.23).

### Experiment 4. EOG recordings

**Figure 4:**
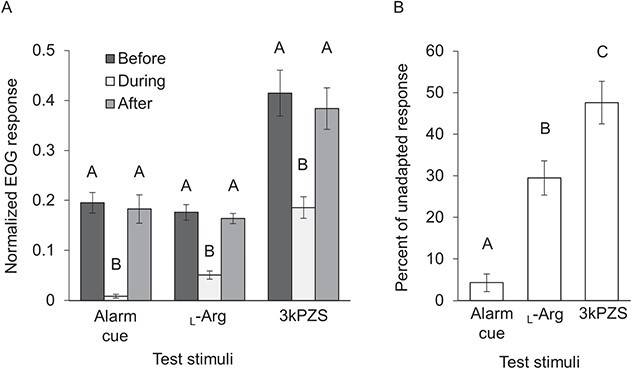
Mean ± SE olfactory responses to alarm cue (1 μl l-1), L-arginine (L-arg; 10–7 M) and 3-keto petromyzonol sulphate (3kPZS; 10–9 M) measured by EOG in immature adult sea lamprey during Experiment #4. (**A**) The normalized EOG response to alarm cue, L-arginine and 3kPZS was measured before, during and after a 15-minute continuous exposure of the olfactory epithelium to dilute alarm cue (1 μl l-1) (*N* = 6). Results from the sensory adaptation experiment show the responses to each test stimuli were reduced during exposure. Responses at time points within a test stimulus that have the same letter are not significantly different from each other (one-way repeated measures ANOVA followed by pairwise paired *t*-test comparisons with Bonferroni adjustment, α = 0.05). Responses to different test stimuli were not compared. (**B**) Continuous exposure of the olfactory epithelium to alarm cue reduced the olfactory response to alarm cue more than L-arginine or 3kPZS (*N* = 6). Test stimuli with the same letter are not significantly different from each other (one-way ANOVA with a Tukey Test, α = 0.05).

The aim of the fourth experiment was to compare the olfactory response to each test stimulus before, during and after a 15-min continuous exposure of the olfactory epithelium to alarm cue. A repeated measures one-way ANOVA revealed the normalized EOG responses to each alarm cue (1 μl l^−1^), _L_-arginine (10^−7^ M) and 3kPZS (10^−9^ M) were different across the three time points within a test stimulus (Alarm cue, F_(2,10)_ = 44.21, *P* < 0.001; _L_-arginine, F_(2,10)_ = 31.87, *P* < 0.001; 3kPZS, F_(2,10)_ = 22.58, *P* < 0.001; Fig. 4A). Post-hoc comparisons (paired *t*-test with Bonferroni adjustments) used to make pairwise comparisons across time points within a stimulus showed the olfactory response to alarm cue during adaptation was less than the unadapted responses (before vs. during, *t*_(5)_ = −9.8, adj. *P* < 0.001; during vs. after, *t*_(5)_ = 6.27, adj. *P* = 0.005). However, the olfactory response to alarm cue after adaptation was not different than the response to alarm cue before (before vs. after, *t*_(5)_ = 0.658, adj. *P* = 1.0), indicating the response to alarm cue was recovered within 15 min or less after flushing the olfactory epithelium with charcoal filtered water. Similar trends were observed when comparing the adapted versus unadapted responses of _L_-arginine (before vs. during, *t*_(5)_ = −8.72, adj. *P* < 0.001; during vs. after, *t*_(5)_ = 9.36, adj. *P* < 0.001; before vs. after, *t*_(5)_ = −0.525, adj. *P* = 1.0) and 3kPZS (before vs. during, *t*_(5)_ = −5.34, adj. *P* = 0.009; during vs. after, *t*_(5)_ = 5.64, adj. *P* = 0.007; before vs. after, *t*_(5)_ = −0.963, adj. *P* = 1.0) with reduced responses recorded to both _L_-arginine and 3kPZS during exposure to alarm cue. Although exposure to alarm cue reduced olfactory responses to all three stimuli tested, the response to alarm cue was reduced more than responses to _L_-arginine or 3kPZS. A one-way ANOVA was conducted and revealed the percent of unadapted responses were different across test stimuli (F_(2,15)_ = 29.54, *P* < 0.001; Fig. 4B). Further, exposure to alarm cue reduced the response to alarm cue (95.7 ± 2.1%, mean ± SE) more than it reduced the response to _L_-arginine (70.5 ± 4.1%, *q* = 6.29, *P* = 0.001) or 3kPZS (52.3 ± 5.1%, *q* = 10.82, *P* < 0.001) according to post-hoc comparisons (Tukey’s HSD test).

## Discussion

The present study suggests sea lamprey exhibit short-term, reversible habituation to a conspecific alarm cue, typified by a response declination involving reduced spatial avoidance of the cue, when exposed continuously for several hours. With continuous immersion in a fixed concentration of the alarm cue, the alarm response was partially attenuated after 2 h and absent after 4 h, consistent with a previous report for sea lamprey (Imre *et al.*, 2017) and notably similar to cane toad (*Bufo marinus*; Hagman and Shine, 2009). Newly observed, the alarm response spontaneously recovered after the cue was eliminated from the environment for 30–60 min. The recorded times of onset and recovery from response attenuation were substantially longer than is typical for the three forms of neuronal adaptation described in vertebrate olfactory systems: short- and long-form sensory adaptation and desensitization (Zufall and Leinders-Zufall, 2000). In EOG studies, direct application of alarm cue to the olfactory epithelium resulted in rapid onset of adaptation, with recovery in 15 min. This was in contrast to the sporadic, repeated exposure that occurred when the animals were allowed to move freely over 5 h. Here, despite frequently encountering and avoiding the alarm cue, we observed no evidence of any change in the avoidance response that would be associated with adaptation, habituation, sensitization or spatial learning. These characteristics implicate habituation as the mechanism of the observed response declination to alarm cue in sea lamprey (per Rankin *et al.*, 2009).

Rankin *et al.*, 2009 (see also McDiarmid *et al.*, 2019) recognize two forms of habituation. Short-term habituation, typically lasting from a few seconds to hours, arises with rapidly repeated stimulation [i.e. a short interstimulus interval (ISI)], whereas long-term habituation may persist for hours to weeks and is more likely to result when ISIs are of relatively long duration. Short-term habituation to persistent cues is considered adaptive, as it allows animals to avoid reacting to uninformative or harmless stimuli. However, long-term habituation to various stimuli that induce avoidance is a well-known problem in management and conservation of animal species (Blumstein, 2016; Greggor *et al.*, 2020). Two features of the application of alarm cue in our study likely resulted in the rapid onset of short-term habituation for animals continuously exposed to the odour. First, the magnitude of the response declination is an inverse function of the ISI, defined as the time between sequential exposures to the stimulus (Rankin and Broster, 1992). In other words, high frequency stimulation of OSNs should lead to more rapid and complete response declination. We applied the cue continuously at a fixed concentration, an ISI = 0. Second, the rapidity of response declination is related to the strength (concentration) of the stimulus. Low concentrations result in more rapid habituation than higher concentrations, and a very strong stimulus may fail to induce habituation. The dilution of alarm cue extract we applied was the minimum necessary to achieve full repellency based on prior laboratory dose–response tests (CM Wagner, unpublished data).

Further support for the hypothesis of short-term habituation is found in the timing of spontaneous recovery of the avoidance behaviour. Habituation is an attentional deficit that dissipates with time. Shorter ISIs are affiliated with more rapid spontaneous recovery of the habituated response (McDiarmid *et al.*, 2019). For example, in a study with *Caenorhabditis elegans*, an ISI of 2 s resulted in full spontaneous recovery after 20–30 min, whereas an ISI of 60 s resulted in partial recovery (≈50% of the unhabituated response) over the same time period (Rankin and Broster, 1992). Here, sea lamprey exhibited no recovery after 30 min of cessation of the stimulus, and full recovery at 60 min (the next measured time step). Finally, sporadic volitional encounter with the odour plume while freely swimming (vs continuous exposure) failed to elicit a response declination after 5 h, despite the animals being observed within the alarm cue plume an average of 28% of the time. Although we did not attempt to analyse individual tracks within the replicate groups, typically, a sea lamprey would enter the alarm cue plume for brief period of 2–20 s, before undertaking a rapid reversal and accelerating away. These sporadic encounters represent a longer ISI, although intervals would be stochastically distributed. Taken together, these findings strongly support a hypothesis of short-term habituation as the underlying mechanism for the behavioural response declination observed during continuous exposure to the alarm cue (vs sensory adaptation or motor fatigue).

The EOG findings clearly indicate that sensory adaptation may also be operating in sea lamprey continuously exposed to alarm cue. However, the EOG study involved direct application of alarm cue to the olfactory epithelium, likely resulting in a large fraction of the alarm cue sensitive OSNs receiving continuous stimulation with a high concentration of the cue. High cue concentration results in more rapid adaptation in vertebrate olfactory neurons (Dalton, 2000). In our laboratory studies, and in the natural circumstance, turbulent advection fractures odour plumes into swirling filaments and packets of odourants as they disperse in the flow (Moore and Crimaldi, 2004). Consequently, an animal holding station a fixed distance downstream from the odour source is likely to encounter a stochastically varying concentration of the alarm cue. Similarly, if the animal moves in response to the encounter with the odour, variation in the cue concentration perceived by the olfactory organ is maintained. Physiologically, these circumstances may maintain a reservoir of unadapted OSNs capable of signalling alarm cue presence to the brain. Finally, sea lampreys do not solely perceive odourants via the main olfactory organ. Solitary chemosensory cells are distributed in the epidermis in association with cutaneous papillae that exhibit responses to a broad array of chemostimulants including food odours and pheromones and may exhibit different adaptation timing vs OSNs (Daghfous *et al.*, 2020). These circumstances further implicate habituation as the primary cause for the reduced behavioural response observed in the lab.

Development of conservation practices after the discovery of useful repellents should involve creation of application practices designed to mitigate sensory and cognitive processes that inhibit the desired response, accounting for the perceptual constraints imposed by the environment (e.g. background sensory noise; Greggor *et al.*, 2014). The sea lamprey alarm cue repellent is most likely to be useful during the annual spawning migration. The sea lamprey undertakes a solitary, nocturnal, non-homing migration from offshore feeding grounds into rivers to spawn, guided by an attractant odour emitted by larvae that reveals the location of suitable spawning habitat (Sorensen *et al.*, 2005; Wagner *et al.*, 2006; Wagner *et al.*, 2009). Because control of the invasive population in the Great Lakes is achieved by applying pesticides to kill the resulting larvae, these annual decisions made by migrants determine the spatial extent of the management challenge in any given year (i.e. the number, size and locations of the rivers requiring future treatment). Consequently, there is considerable interest in manipulating movement decisions in rivers to either remove migrants prior to spawning (e.g. guide them into fishing devices) or guide them into fewer tributaries to reduce the overall spatial extent of the infestation prior to treatment (Li *et al.*, 2007; Marsden and Siefkes, 2019). Coincidentally, the riverine migration is also a time of great interest for the conservation of native parasitic lampreys, including the Pacific (*Entosphenus tridentatus*), European river (*Lampetra fluviatilis*) and sea lamprey in its native range (Igoe *et al.*, 2004; Clemens *et al.*, 2017). The principal threat to these taxa during migration is the placement of barriers to migration, and conservation managers are interested in the ability to guide migrants into fish passage devices (Maitland *et al.*, 2015; Pereira *et al.*, 2019).

Although the nocturnal movement schedule should allow any short-term habituated or adapted sea lamprey to recover the alarm response on the following day, continuous application of the alarm cue at night is contraindicated. In rivers, reported ground speeds for migrating sea lamprey range from 29.3 to 45.6 body lengths per min (Almeida *et al.*, 2002; Quintella *et al.*, 2009; Castro-Santos *et al.*, 2017), equating to a movement range of 2.1–3.3 km in 4 h for a continuously cruising individual of 300-mm body length (typical of land-locked populations). This is likely an overestimate, as sea lamprey exhibit periods of rest during upstream movement on a given night (Almeida *et al.*, 2002). However, encounter with the alarm cue while migrating upstream may also elicit an increase in ground speed (Luhring *et al.*, 2016). The alarm cue will fully mix in the river some distance downstream of the application point and persist until chemical breakdown or dilution from downstream tributaries, a distance we expect to be considerably longer than 5 km in most rivers. Consequently, migrants are likely to habituate prior to encounter with the application site if pumped continuously into the flow.

Our current understanding of habituation in vertebrates offers three potential remedies (per Rankin *et al.*, 2009; McDiarmid *et al.*, 2019): ‘pulsing’ the stimulus input (increasing ISI), increasing the concentration or introducing a dishabituating stimulus. First, increasing the ISI (pulsing the odour on/off) will increase the time until a response decrement occurs, as evident in the results of Experiment #3. Pulses would have to be modulated such that full mixing is avoided for the desired distance downstream of the application point. One consequence of a relatively long ISI (minutes) would be an increased chance of animals failing to detect the odour in the inter-pulse periods near the application site. Second, the concentration of the odour may be increased to reduce or eliminate the onset of habituation. Little is known about the onset of habituation to high concentration odours in fishes over periods longer than typical laboratory experiments (i.e. minutes to hours) suggesting further research would be needed in the development phase of any alarm cue repellent. Third, unlike sensory adaptation or motor fatigue, habituation involves modification of an animal’s attentional response to a stimulus. Consequently, the presentation of a strong stimulus that differs from the habituated stimulus (e.g. loud noise, light flash) induces spontaneous recovery of the original stimulus response termed dishabituation (e.g. electric fish *Gnathonemus petersii*, Post and von der Emde, 1999; marine mollusc *Aplysia californica*, Hawkins *et al.*, 2006; Lepidopteran insect *Trichoplusia ni*, Akhtar *et al.*, 2003).

Whether long-term exposure to alarm cue that is pulsed (longer ISIs) or pumped continuously at high concentration would bring about long-term habituation is unknown. Imre *et al.* 2016 failed to observe habituation to an alarm cue in sea lamprey when subjects were exposed four or eight times in the 24 h prior to evaluation of their responses. However, chub (*Squalius cephalus*; Krause, 1993a) and common dace (*Leuciscus leuciscus;*  Krause, 1993b*)* exhibited a response declination to their alarm cues when exposed twice daily for 14 (chub) or 10 (dace) consecutive days. These studies did not examine spontaneous recovery of the alarm response. Interestingly, long-term habituation to alarm cue may help explain the observation that fishes in high predation environments exhibit higher response thresholds to alarm cue vs those in low predation environments (e.g. Brown *et al.*, 2006; Mirza *et al.*, 2006). For migratory fishes, brief periods of exposure prior to capture or passage through the application site may prevent serious impacts to conservation and management objectives. Where the goal is to create continuous avoidance of an area for resident fishes (e.g. water intake structures), long-term habituation would pose a problem. The dishabituation phenomenon may imply a solution (e.g. presentation of another strong stimulus to dishabituate resident fishes to the alarm cue(s) in use). However, as the process of identifying the chemical constituents in a fish pheromone can be costly and time-consuming, development of alarm cue based repellents may be recommended only in cases where the benefits to conservation are substantial. Alternatively, an alarm cue may prove effective as a species-specific dishabituating stimulus for non-chemical deterrents that represent conditioned stimuli (e.g. sound barriers for invasive carps; Dennis and Sorensen, 2020), requiring far less cue that may be harvested from animal tissue. In other words, the alarm cue for a species that proves particularly problematic may be used to dishabituate the local population to another more cost-effective deterrent (sound barriers, bubble curtains, light barriers, etc.), extending the useful life of the management practice. Further, fishes and other aquatic organisms can learn to associate novel environmental (unconditioned) stimuli with predation risk, when a deterrent stimulus is periodically paired with the alarm cue, creating more consistent avoidance through reinforcement (Greggor *et al.*, 2020).

The present study supports the hypothesis that short-term habituation underlies the response declination observed in the alarm response in sea lamprey when exposed to a continuous application of alarm cue. Although adaptive, this physiological phenomenon produces substantial challenges for the development of a species-specific repellent. Fortunately, a large body of research into olfactory habituation provides potential remedies in the form of application practices. Further development of this approach should include the following: (i) examination of the effect of longer duration stimulus pulses (e.g. signals that continue for sec to min), (ii) interactions between pulse durations ISIs on the persistence of behavioural responses and (iii) the incorporation of dishabituating stimuli to achieve more effective conservation practices.

## Data Availability

The data underlying this article will be shared on reasonable request to the corresponding author.

## References

[ref1] Akhtar Y, Rankin CH, Isman MB (2003) Decreased response to feeding deterrents following prolonged exposure in larvae of a generalist herbivore, Trichoplusia ni (Lepidoptera: Nocturidae). J Insect Behav 16: 811–831.

[ref2] Almeida PR, Quintella BR, Dias NM (2002) Movement of radio-tagged anadromous sea lamprey during spawning migration in the River Mondego (Portugal). Hydrobiologia 483: 1–8.

[ref3] Bals JB, Wagner CM (2012) Behavioral responses of sea lamprey (*Petromyzon marinus*) to a putative alarm cue derived from conspecific and heterospecific sources. Behaviour 149: 901–923.

[ref4] Blumstein DT (2016) Habituation and sensitization: new thoughts about old ideas. Anim Behav 120: 255–262.

[ref5] Brant C (2019) Great Lakes Sea Lamprey: The 70 Year War on a Biological Invader. University of Michigan Press, Ann Arbor, MI

[ref6] Brown GE, Rive AC, Ferrari MCO, Chivers DP (2006) The dynamic nature of antipredator behavior: prey fish integrate threat-sensitive antipredator responses within background levels of predation risk. Behav Ecol Sociobiol 61: 9–16.

[ref7] Buchinger TJ, Scott AM, Fissette SD, Brant CO, Huertas M, Li K, Johnson NS, Li W (2020) A pheromone antagonist liberates female sea lamprey from a sensory trap to enable reliable communication. Proc Natl Acad Sci U S A 117: 7284–7289. 32184327 10.1073/pnas.1921394117 PMC7132252

[ref8] Butler SE, Porreca AP, Collins SF, Freedman JA, Parkos JJ III, Diana MJ, Wahl DH (2019) Does fish hearding enhance capture rates and detection of invasive bigheaded carp? Biol Invasions 21:775–785.

[ref9] Byford GJ, Hume JB, Moser ML, Wagner CM (2016) Do native Pacific lamprey and invasive sea lamprey share an alarm cue? Implications for use of a natural repellent to guide imperiled Pacific lamprey into fishways. N Am J Fish Manag 36: 1090–1096.

[ref10] Castro-Santos T, Haro A (2010) Fish guidance and passage at barriers. In P Domenici, BG Kapoor, eds, Fish Locomotion: An Eco-ethological Perspective. CRC Press, USA, pp. 62–89.

[ref11] Castro-Santos T, Shi X, Haro A (2017) Migratory behavior of adult sea lamprey and cumulative passage performance through four fishways. Can J Fish Aquat Sci. 74: 790–800.

[ref12] Chivers DP, Dixson DL, White JR, McCormick MI, Ferrari MCO (2013) Degradation of chemical alarm cues and assessment of risk throughout the day. Ecol Evol 3: 3925–3934. 24198950 10.1002/ece3.760 PMC3810885

[ref13] Clemens BJ, Beamish RJ, Coates KC, Docker MF, Dunham JB, Gray AE, Hess JE, Jolley JC, Lampman RT, McIlraith BJ et al. (2017) Conservation challenges and research needs for Pacific lamprey in the Columbia River basin. Fisheries 42: 268–280.

[ref14] Daghfous G, Auclair F, Blumenthal F, Suntres T, Lamarre-Bourret J, Mansouri M, Sielinski B, Dubuc R (2020) Sensory cutaneous papillae in the sea lamprey (Petromyzon marinus): I. J Comp Neurol Psychol 528: 664–686. 10.1002/cne.24787 31605382

[ref15] Dalesman S, Rundle SD, Cotton PA (2007) Predator regime influences innate anti-predator behaviour in the freshwater gastropod *Lymnaea stagnalis*. Freshw Biol 52: 2134–2140.

[ref16] Dalton P (2000) Psychophysical and behavioral characteristics of olfactory adaptation. Chem Senses 25: 487–492. 10944515 10.1093/chemse/25.4.487

[ref17] Dennis CE, Sorensen PW (2020) Common carp are initially repelled by a broadband outboard motor sound in a lock chamber but habituate rapidly. N Am J Fish Manag 40: 1499–1509.

[ref18] Di Rocco RT, Johnson NS, Brege L, Imre I, Brown GE (2016) Sea lamprey avoid areas scented with conspecific tissue extract in Michigan streams. Aquacult Fish Manag 23: 548–560.

[ref19] Døving KB, Lastein S (2009) The alarm reaction in fishes—odorants, modulations of responses, neural pathways. Ann N Y Acad Sci 1170: 413–423. 19686169 10.1111/j.1749-6632.2009.04111.x

[ref20] Farnsley S, Kuhajda B, George A, Klug H (2018) Learning to overcome a lack of evolutionary history: can an endangered fish learn to fear an introduced predator? Front Ecol Evol 6: 214.

[ref21] Ferrari MCO, Wisenden BD, Chivers DP (2010) Chemical ecology of predator-prey interactions in aquatic ecosystems: a review and prospectus. Can J Zool 88: 698–724.

[ref22] von Frisch K (1938) Zur psychologie des Fische-Schwarmes. Naturwissenschaften 26: 601–606.

[ref23] von Frisch K (1941) Die Bedeutung des Geruchsinnes im Leben der Fische. Naturwissenschaften 29: 321–333.

[ref24] Gaynor KM, Brown JS, Middleton AD, Power ME, Brashares JS (2019) Landscapes of fear: spatial patterns of risk perception and response. Trends Ecol Evol 34: 355–368. 30745252 10.1016/j.tree.2019.01.004

[ref25] Greggor AL, Berger-Tal O, Blumstein DT (2020) The rules of attraction: the necessary role of animal cognition in explaining conservation failures and successes. Ann Rev Ecol Syst 51: 483–503.

[ref26] Greggor AL, Clayton NS, Phalan B, Thornton A (2014) Comparative cognition for conservationists. Trends Ecol Evol 29: 489–495. 25043737 10.1016/j.tree.2014.06.004 PMC4153814

[ref27] Hagman M, Shine R (2009) Factors influencing responses to alarm pheromone by larvae of invasive cane toads. J Chem Ecol 35: 265. 19184225 10.1007/s10886-009-9592-x

[ref28] Hamdani EH, Døving KB (2002) The alarm reaction in Crucian carp is mediated by olfactory neurons with long dendrites. Chem Senses 27: 395–398. 12006379 10.1093/chemse/27.4.395

[ref29] Hamdani EH, Døving KB (2007) The functional organization of the fish olfactory system. Prog Neurobiol 82: 80–86. 17433527 10.1016/j.pneurobio.2007.02.007

[ref30] Hawkins RD, Cohen TE, Kandel ER (2006) Dishabituation in Aplysia can involve either reversal of habituation or superimposed sensitization. Learn Mem 13: 397–403. 16705138 10.1101/lm.49706 PMC1475823

[ref31] Hazlett BA, McLay C (2005) Responses of the crab *Heterozius rotundifrons* to heterospecific chemical alarm cues: phylogeny vs. ecological overlap. J Chem Ecol 31: 671–677. 15898508 10.1007/s10886-005-2054-1

[ref32] Hume JB, Luhring TM, Wagner CM (2020) Push, pull, or push-pull? A predation cue better guides migrating sea lamprey towards capture devices than a mating pheromone during the reproductive migration. Biol Invasions 22: 2129–2142.

[ref33] Hume JB, Meckley TD, Johnson NS, Luhring TM, Siefkes MJ, Wagner CM (2015) The application of an alarm cue in a push-pull configuration hastens arrival of invasive sea lamprey (*Petromyzon marinus*) at a trapping location. Can J Fish Aquat Sci 72: 1799–1806.

[ref34] Hume JB, Wagner CM (2018) A death in the family: sea lamprey (*Petromyzon marinus*) avoidance of confamilial alarm cues diminishes with phylogenetic distance. Ecol Evol 8: 3751–3762. 29686855 10.1002/ece3.3930 PMC5901161

[ref35] Igoe F, Quigley DTG, Marnell F, Meskell E, O’Connor W, Byrne C (2004) The sea lamprey *Petromyzon marinus* (L.), river lamprey *Lampetra fluviatilis* (L.) and brook lamprey *Lampetra planeri* (Bloch) in Ireland: general biology, ecology, distribution and status with recommendations for conservation. Proceedings of the Royal Irish Academy, Section B Biol Geol Chem Sci 104B: 43–56.

[ref36] Imre I, Brown GE, Bergstedt RA, McDonald R (2010) Use of chemosensory cues as repellents for sea lamprey: potential directions for population management. J Great Lakes Res 36: 790–793.

[ref37a] Imre I, Di Rocco RT, Brown GE, Johnson NS, Johnson NS (2016) Habituation of adult sea lamprey repeatedly exposed to damage-released alarm and predator cues. Env Biol Fishes 99: 613–620.

[ref37] Imre I, Di Rocco RT, McClure H, Johnson NS, Brown GE (2017) Migratory-stage sea lamprey *Petromyzon marinus* stop responding to conspecific damage-released alarm cues after 4 h of continuous exposure in laboratory conditions. J Fish Biol 90: 1297–1304. 27957739 10.1111/jfb.13231

[ref38] Krause J (1993a) Transmission of fright reaction between different species of fish. Behaviour 127: 37–48.

[ref39] Krause J (1993b) The effect of ‘Schreckstoff’ on the shoaling behavior of the minnow: a test of Hamilton’s selfish herd theory. Anim Behav 45: 1019–1024.

[ref40] Kurahashi T, Menini A (1997) Mechanism of odorant adaptation in the olfactory receptor cell. Nature 385: 725–729. 9034189 10.1038/385725a0

[ref41] Li W, Scott AP, Siefkes MJ, Yan H, Liu Q, Yun S-S (2002) Bile acid secreted by male sea lamprey that acts as a sex pheromone. Science 296: 138–141. 11935026 10.1126/science.1067797

[ref42] Li W, Twohey MB, Jones ML, Wagner CM (2007) Research to guide use of pheromones to control sea lamprey. J Great Lakes Res 33: 70–86.

[ref43] Lima SL, Dill LM (1990) Behavioral decisions made under the risk of predation: a review and prospectus. Can J Zool 68: 619–640.

[ref44] Luhring TM, Meckley TD, Johnson NS, Siefkes MJ, Hume JB, Wagner CM (2016) A semelparous fish continues upstream migration when exposed to alarm cue, but adjusts movement speed and timing. Anim Behav 121: 41–51.

[ref45] Maitland PS, Renaud CB, Quintella BR, Close DA, Docker MF (2015) Conservation of native lampreys. In MF Docker, ed, Lampreys: Biology, Conservation and Control, Vol. 1. Springer, Dordrecht, pp.375–428.

[ref46] Marsden JE, Siefkes MJ (2019) Control of invasive sea lamprey in the Great Lakes, Lake Champlain, and Finger Lakes of New York. In MF Docker, ed, Lampreys: Biology, Conservation and Control, Vol. 2. Springer, Dordrecht, pp. 411–479.

[ref47] McDiarmid TA, Yu AJ, Rankin CH (2019) Habituation is more than learning to ignore: multiple mechanisms serve to facilitate shifts in behavioral strategy. Bioessays 41: 1900077. 10.1002/bies.201900077 31429094

[ref48] Mirza RS, Mathis A, Chivers DP (2006) Does temporal variation in predation risk influence the intensity of antipredator responses? A test of the risk allocation hypothesis. Ethology 112: 44–51.

[ref49] Mitchell MD, Cowman PF, McCormick MI (2012) Chemical alarm cues are conserved within the coral reef fish family Pomacentridae. PLoS One 7: e47428. 23094047 10.1371/journal.pone.0047428 PMC3475700

[ref50] Moore P, Crimaldi J (2004) Odor landscapes and animal behavior: tracking odor plumes in different physical worlds. J Mar Syst 49: 55–64.

[ref51] Moser ML, Corbett SC, Keefer ML, Frick KE, Lopez-Johnston S, Caudill CC (2019) Novel fishway entrance modifications for Pacific lamprey. J Ecohydraul 4: 71–84.

[ref52] Moser ML, Keefer ML, Pennington HT, Ogden DA, Simonson JE (2011) Development of Pacific lamprey fishways at a hydropower dam. Fish Manag Ecol 18: 190–200.

[ref53] Moser ML, Matter AL, Stuenhrenberg LC, Bjornn TC (2002) Use of an extensive radio receiver network to document Pacific lamprey (*Lampetra tridentata*) entrance efficiency at fishways in the lower Columbia River, USA. Hydrobiologia 483: 45–53.

[ref54] Noatch MR, Suski CD (2012) Non-physical barriers to deter fish movements. Environ Rev 20: 71–82.

[ref55] Pereira E, Cardoso GR, Quintella BR, Mateus CS, Alexandre CM, Oliveira RL, Belo AF, Telhado A, Quadrado MF, Batista CM et al. (2019) Proposals for optimizing sea lamprey passage through a vertical-slot fishway. Ecol Eng 12: e2087.

[ref56] Pinheiro J, Bates D, Deb Roy S, Sarkar D, the R Development Core Team (2015) Package nlme: linear and nonlinear mixed effects models. R Package version 3.1–103.

[ref57] Porter LL, Hayes MC, Jackson AD, Burke BJ, Moser ML, Wagner RS (2017) Behavioral responses of Pacific lamprey to alarm cues. J Fish Wildl Manag 8: 101–113.

[ref58] Post M, von der Emde G (1999) The “novelty response” in an electric fish: response properties and habituation. Physiol Behav 68: 115–128. 10627070 10.1016/s0031-9384(99)00153-5

[ref59] Preisser EL, Bolnick DI (2008) The many faces of fear: comparing pathways and impacts of nonconsumptive predator effects on prey populations. PLoS One 3: e2465. 18560575 10.1371/journal.pone.0002465 PMC2409076

[ref60] Quintella BR, Póvia I, Almeida PR (2009) Swimming behavior of upriver migrating sea lamprey assess by electromyogram telemetry. J Appl Ichthyol 25: 46–54.

[ref61] Rankin CH, Abrams T, Barry RJ, Bhatnagar S, Clayton DF, Colombo J, Coppola G, Geyer MA, Glanzman DL, Marsland S et al. (2009) Habituation revisited: an updated and revised description of the behavioral characteristics of habituation. Neurobiol Learn Mem 92: 135–138. 18854219 10.1016/j.nlm.2008.09.012 PMC2754195

[ref62] Rankin CH, Broster BS (1992) Factors affecting habituation and recovery from habituation in the nematode *Caenorhabditis elegans*. Behav Neurosci 106: 239–249. 1590951 10.1037//0735-7044.106.2.239

[ref63] Sabal MC, Boyce MS, Chaprentier CL, Furey NB, Luhring TM, Martin HW, Melnychuk MC, Srygley RB, Wagner CM, Wirsing AJ et al. (2021) Predation landscapes influence migratory prey ecology and evolution. Trends Ecol Evol 36. 10.1016/j.tree.2021.04.010 33994219

[ref64] Schoeppner NM, Relyea RA (2009) When should prey respond to consumed heterospecifics? Testing hypotheses of perceived risk. Copeia 2009: 190–194.

[ref65] Siefkes MJ, Li W (2004) Electrophysiological evidence for detection and discrimination of pheromonal bile acids by the olfactory epithelium of female sea lampreys (*Petromyzon marinus*). J Comp Physiol A 190: 193–199. 10.1007/s00359-003-0484-1 14689221

[ref66] Sih A, Ziemba I, Harding KC (2000) New insights on how temporal variation in predation risk shapes prey behavior. Trends Ecol Evol 15: 3–4. 10603494 10.1016/s0169-5347(99)01766-8

[ref67] Sorensen PW (2015) Applications of pheromones in invasive fish control and fishery conservation. In PW Sorensen, BD Wisenden, eds, Fish Pheromones and Related Cues. Wiley-Blackwell Press, Hoboken, New Jersey, pp. 255–268.

[ref68] Sorensen PW, Fine JM, Dvornikovs V, Jeffrey CS, Shao F, Wang J, Vrieze LA, Anderson KR, Hoye TR (2005) Mixture of new sulfated steroids functions as a migratory pheromone in the sea lamprey. Chem Biol 1: 324–328. 10.1038/nchembio739 16408070

[ref69] Sorensen PW, Johnson NS (2016) Theory and application of semiochemicals in nuisance fish control. J Chem Ecol 42: 698–715. 27417504 10.1007/s10886-016-0729-4

[ref70] Wagner CM, Jones ML, Twohey MB, Sorensen PW (2006) A field test verifies that pheromones can be useful for sea lamprey (*Petromyzon marinus*) control in the Great Lakes. Can J Fish Aquat Sci 63:475–479.

[ref71] Wagner CM, Stroud EM, Meckley TD (2011) A deathly odor suggests a new sustainable tool for controlling a costly invasive species. Can J Fish Aquat Sci 68: 1157–1160.

[ref72] Wagner CM, Twohey MB, Fine JM (2009) Conspecific cueing in the sea lamprey: do reproductive migrations consistently follow the most intense larval odor? Anim Behav 78: 593–599.

[ref73] Wilson DA, Linster C (2008) Neurobiology of a simple memory. J Neurophysiol 100: 2–7. 18463176 10.1152/jn.90479.2008

[ref74] Wisenden BD (2015) Chemical cues that indicate risk of predation. In PW Sorensen, BD Wisenden, eds, Fish pheromones and related cues. Wiley-Blackwell Press, Hoboken, New Jersey, pp. 131–148.

[ref75] Wisenden BD, Rugg ML, Korpi NL, Fuselier LC (2009) Lab and field estimates of active time of chemical alarm cues of a cyprinid fish and an amphipod crustacean. Behaviour 146: 1423–1442.

[ref76] Zufall F, Leinders-Zufall T (2000) The cellular and molecular basis of odor adaptation. Chem Senses 25: 473–481. 10944513 10.1093/chemse/25.4.473

